# Establishment of an Individualized Predictive Model to Reduce the Core Number for Systematic Prostate Biopsy: A Dual Center Study Based on Stratification of the Disease Risk Score

**DOI:** 10.3389/fonc.2021.831603

**Published:** 2022-02-14

**Authors:** Zeyu Chen, Min Qu, Xianqi Shen, Shaoqin Jiang, Wenhui Zhang, Jin Ji, Yan Wang, Jili Zhang, Zhenlin Chen, Lu Lin, Mengqiang Li, Cheng Wu, Xu Gao

**Affiliations:** ^1^ Department of Urology, Shanghai Changhai Hospital, Second Military Medical University, Shanghai, China; ^2^ Department of Urology, Fujian Union Hospital, Fujian Medical University, Fuzhou, China; ^3^ Department of Health Statistics, Second Military Medical University, Shanghai, China

**Keywords:** prostate biopsy, multiparameter MRI, PI-RADS, multicenter study, predictive models

## Abstract

**Purpose:**

To establish an individualized prostate biopsy model that reduces unnecessary biopsy cores based on multiparameter MRI (mpMRI).

**Materials and Methods:**

This retrospective, non-inferiority dual-center study retrospectively included 609 patients from the Changhai Hospital from June 2017 to November 2020 and 431 patients from the Fujian Union Hospital between 2014 and 2019. Clinical, radiological, and pathological data were analyzed. Data from the Changhai Hospital were used for modeling by calculating the patients’ disease risk scores. Data from the Fujian Union Hospital were used for external verification.

**Results:**

Based on the data of 609 patients from the Changhai Hospital, we divided the patients evenly into five layers according to the disease risk score. The area under the receiver operating characteristic (ROC) curve (AUC) with 95% confidence intervals (CI) was analyzed. Twelve-core systemic biopsy (12-SBx) was used as the reference standard. The SBx cores from each layer were reduced to 9, 6, 5, 4, and 4. The data of 279 patients with benign pathological results from the Fujian Union Hospital were incorporated into the model. No patients were in the first layer. The accuracies of the models for the other layers were 88, 96.43, 94.87, and 94.59%. The accuracy of each layer would be increased to 96, 100, 100, and 97.30% if the diagnosis of non-clinically significant prostate cancer was excluded.

**Conclusions:**

In this study, we established an individualized biopsy model using data from a dual center. The results showed great accuracy of the model, indicating its future clinical application.

## Introduction

Prostate biopsy is the standard procedure for tissue acquisition for pathological diagnosis. Since the 1980s, when TRUS-guided 6-core systematic biopsy was proposed by Hodge et al. (1), it was found to have a 33% misdiagnosis rate for Pca (2). Thus, 8-, 10-, or 12-core and even saturation biopsies are recommended to improve the detection rate, even though they increase the risk of rectal bleeding, urinary tract infection, erectile dysfunction, and other complications (3, 4).

Since MRI-guided prostate biopsy was first performed by D’Amico in 2000 (5), it has been proven to detect more csPCa with fewer biopsy cores than system biopsy by high-quality research (6). It is still unclear how to decrease the number of cores under the condition that more csPCa is diagnosed (7, 8). Because mpMRI inter-reader reproducibility remains moderate at best (9, 10), the accuracy and reproducibility of targeted biopsy still need to be improved (11), and the optimal core number and site for MRI-targeted biopsy have not been clearly elucidated (12). Furthermore, the NVP of MRI-guided prostate biopsy is unstable (13). Thus, the EAU Guidelines recommend combining targeted and systematic biopsy in patients who are naïve in biopsy when mpMRI is positive (i.e., PI-RADS ≥3) (14), which will increase the risk of complications (15).

Based on the existence of many unstable factors in MRI-guided prostate biopsy, an alternative approach is to reduce the number of cores on systemic biopsy. Current research has focused on specific factors, such as prostate volume or PSA level, to reduce the cores of systematic biopsy (16), or analyzed different hypothetical sampling schemes when compared with targeted biopsy plus 12-SBx (17). Fewer studies have reported the reduction of cores after individualization of patients according to the location of suspicious lesions on MRI, and a variety of factors are not yet sufficient.

Therefore, we performed a dual-center, non-inferiority study to establish an individualized predictive model to optimize prostate biopsy. The model can plan the biopsy location by reducing the cores after comprehensively incorporating the basic information, tumor indicators, mpMRI, and other related patient factors. We circumvented the deficiencies of similar research, and for the first time combined multiple factors into one completed individualized analysis of basic patient information. On the other hand, we made a website for model use, which is convenient for clinics. At the same time, this website can input information about the nodule position of the patient, which completes the individualized analysis of mpMRI information.

## Materials and Methods

### Participants

**Figure 1:**
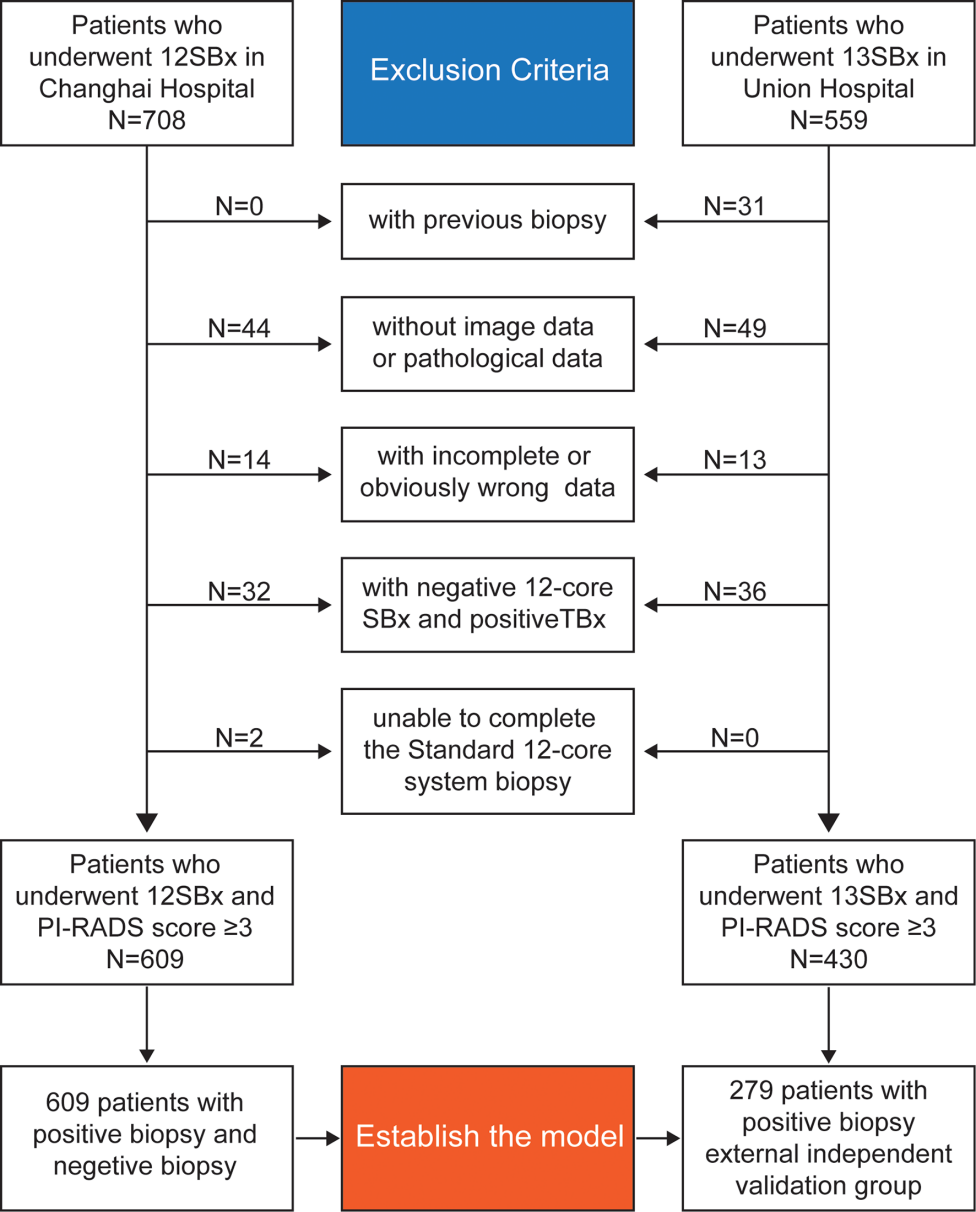
Diagram for inclusion of patients in the study.

This study retrospectively analyzed patients who underwent 12-core (or 13-core) transrectal US-guided prostate biopsy for suspected PCa with a PI-RADS score ≥3 at the Changhai Hospital and the Fujian Union Hospital. Diagram for inclusion of patients in the study are shown in **Figure 1**.

The inclusion criteria were: (1) indications for biopsy and (2) abnormal nodules revealed on mpMRI (PI-RADS score ≥3).

The exclusion criteria were: (1) previous biopsy; (2) inability to complete the standard 12-core system biopsy; (3) cases with antiandrogen therapy; (4) negative 12-core system biopsy and positive MRI-guided prostate biopsy; and (5) incomplete or obviously wrong data.

A total of 609 patients were enrolled in the Changhai Hospital to establish the model and 279 patients with positive biopsy in the Fujian Union Hospital were enrolled as an external independent validation group.

**Figure 2:**
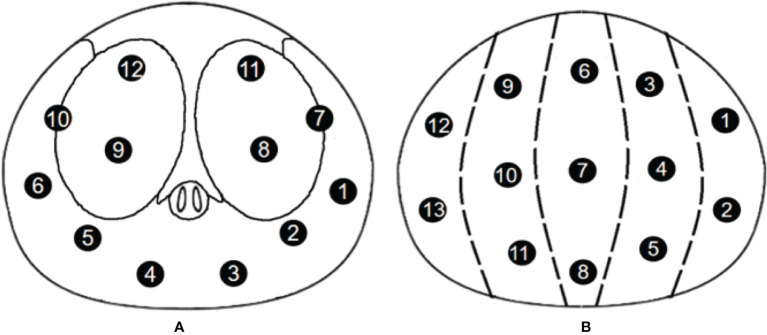
Biopsy point distribution diagram for the two centers **(A)** 12-core, Changhai Hospital; **(B)** 13-core, Fujian Union Hospital).

Patients in the Changhai Hospital underwent 12-SBx (as shown in **Figure 2A**), and some patients underwent MRI-guided prostate biopsy. Patients in the Fujian Union Hospital underwent 13-SBx (as shown in **Figure 2B**), and some patients underwent MRI-guided prostate biopsy.

### mpMRI and Scan Protocol

mpMRI examinations were performed using a 3.0-T system (Magnetom Skyra, Siemens Medical Solutions, Erlangen, Germany) with an 18-channel phased-array coil, before biopsy. The sequences of examination mainly included T2-weighted imaging (T2WI), diffusion-weighted imaging (DWI), and dynamic contrast-enhanced imaging. Two radiologists with >10 years of experience in MRI evaluated and scored the images according to the Prostate Imaging Reporting and Data System version 2 criteria (18). Next, three diameters of the prostate and the smallest diameter of the suspicious lesion were measured and calculated using mpMRI on the T2WI sequence.

### Prostate Biopsy Method

The entire prostate biopsy processes were completed by a surgeon with more than five years of experience. Preoperative routine examinations included routine blood tests, coagulation function, liver and kidney function, urine routine, fecal routine, and serum PSA concentration. If patients used aspirin, warfarin, and other anticoagulants, they were requested to stop using the drugs for 2 weeks. A cleansing enema was also performed on patients in the lithotomy position, and the perineum and perianal region were disinfected with 0.5% iodophor. A novel perineal nerve block approach (19) was adopted with 5% lidocaine.

An ultrasound probe was inserted through the anus to measure the three diameters of the prostate followed by a 12-needle system biopsy. A biopsy point distribution diagram for the two centers is shown in **Figure 2**. Patients in the Changhai Hospital underwent 12-SBx (as shown in **Figure 2A**), and some patients underwent MRI-guided prostate biopsy. Patients in the Fujian Union Hospital underwent 13-SBx (as shown in **Figure 2B**), and some patients underwent MRI-guided prostate biopsy.

After the biopsy, for bleeding patients, an iodophor gauze was placed around the perineum. The tissues were fixed in 10% formalin.

### The Simulated Process of Reducing the Number of Cores

Two clinicians (Doctors A and B) with >5 years of experience in mpMRI complete the simulated process of reducing the number of cores. Doctor A knew the pathological results of each patient (accurate to the pathology of each core), and Doctor B knew the mpMRI data of each patient, and the biopsy point distribution diagram.

**Figure 3:**
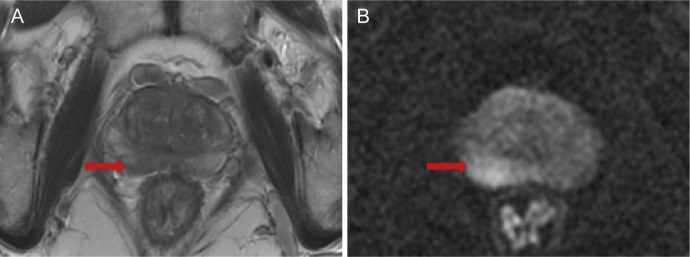
**(A)** T2-weighted imaging (T2WI); **(B)** diffusion-weighted imaging (DWI).

**Figure 4:**
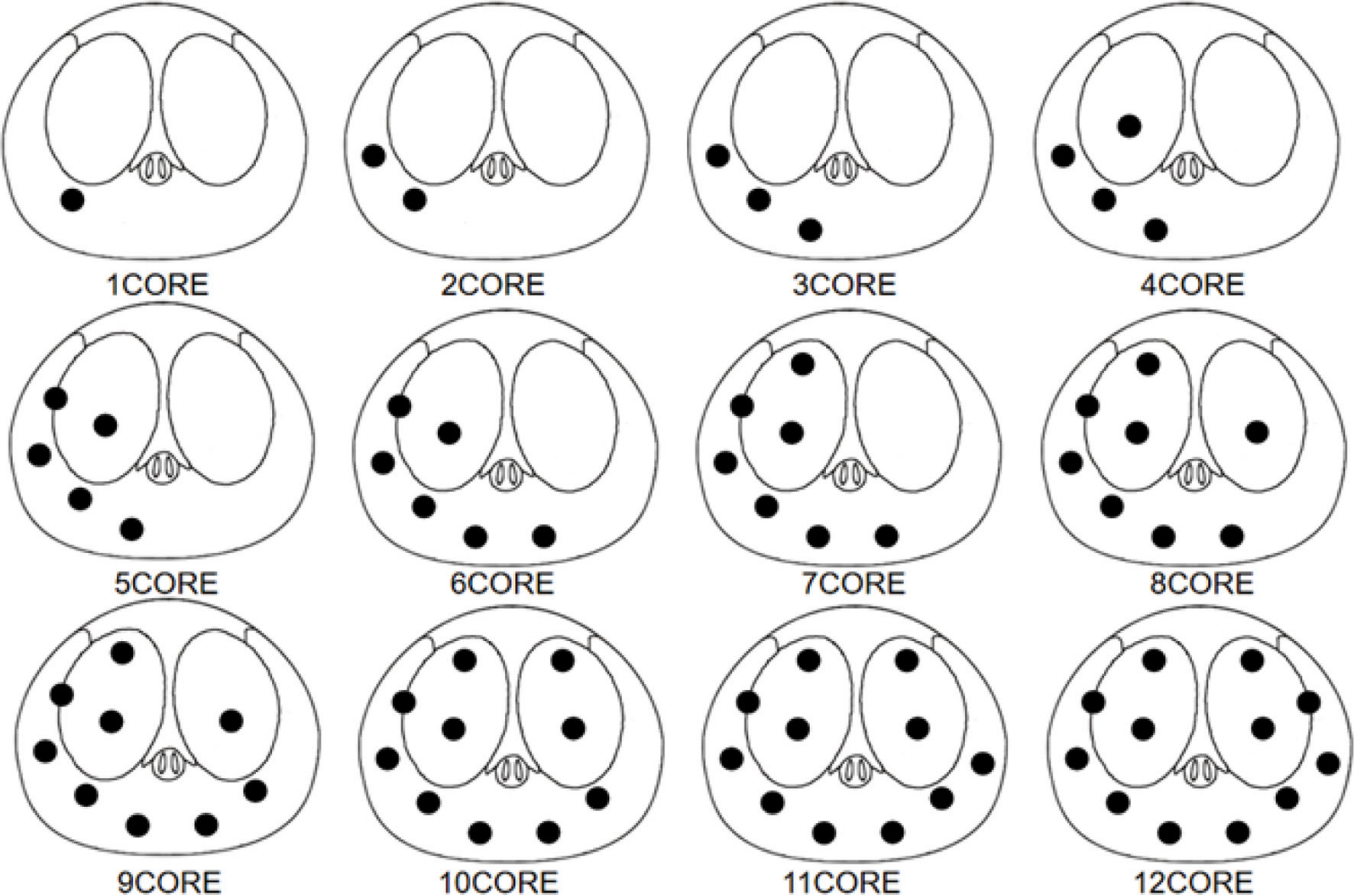
Biopsy point distribution diagram for the 1-core to 12-core methods.

Doctor B judged the location of the most suspicious lesion based on the mpMRI based on the distance between the center of the lesion location and each systemic biopsy core. When one core was taken, the closest core to the lesion was recorded; when two cores were taken, the two cores closest to the lesion were recorded; this simulated process was restored to 12 cores. For example, if the lesion was located in the right peripheral zone of the base zone (as shown in **Figure 3**), the simulated system biopsy process was restored 12 times (**Figure 4**).

Doctor B did not know the pathological results of each simulated system biopsy process; if there were two or more suspicious lesions, Doctor B independently determined the location of the most suspicious lesions based on clinical experience, and Doctor A recorded the negative or positive result of simulated biopsy under different numbers of biopsy cores.

### Statistical Analysis

Measurement data were tested whether they followed normal distribution. Measurement data with normal distribution and equal variance was described by mean ± standard deviation (SD), and independent samples t-test was used for comparison between two independent samples; measurement data with non-normal distribution or unequal variance was described by Median (Q1–Q3), and Mann–Whitney test was used. The categorical data was described by n (%), and Pearson chi-square test or Fisher’s exact test was used to test the difference between the two groups.

We used a Disease Risk Score (DRS) to measure the risk of prostate cancer for each participation. DRS is a comprehensive index based on all covariates, which is defined as the probability of occurrence of final events under the condition of certain covariates in the model. Logistic regression was used to calculate the DRS. Then the participation were equally divided into 5 layers according to the percentiles of their DRS, that is, Min–P20, P20–P40, P40–P60, P60–P80, and P80–Max. [Distribution range of Disease Risk Score (DRS) is shown in the **Supplementary Table 14**].

We calculated the detection rate of prostate cancer in different layers. Moreover, the sensitivity, negative prediction value, accuracy and area under the ROC curve (AUC) with 95%CI were analyzed, and the values were compared with those of the 12-core systematic biopsy results to get the most suitable number of biopsy cores for each layer(the lower boundary of the two-sided 95%CI ≥95%). McNemar’s test was used to compare the detection rate between 12-core systematic biopsy and 1-core to 11-core method.

We validate the constructed DRS layers and their most suitable number of biopsy cores with external data. We used the disease risk score model of Changhai Hospital to calculate the DRS of patients with positive prostate puncture in the Fujian Union Hospital, then, stratified the patients into 5 layers according to the stratification method of Changhai Hospital. The detection rate of prostate cancer of the patients and its 95% CI in each layer was calculated.

After validation, we also developed a web-based visualization tool for clinical application (website: https://daringsky.shinyapps.io/prediction_v2/).

Statistical significance was defined as two-sided p-value of <0.05. Statistical analyses were performed using SPSS v24.0 (SPSS Inc., Chicago, IL, USA).

## Results

### Predictive Model Using the Disease Risk Score Based on Data From the Changhai Cohort

#### Patients’ Baseline Characteristics

There was no significant statistical difference in the BMI of the patients (p = 0.482) and longest diameter of lesions (p = 0.138), while patients with PCa were older, had higher PSA, larger prostate volume, and higher PI-RADS scores (all p <0.05) (as shown in **Table 1**).

**Table 1:** Characteristics of patients in Changhai Hospital.

	total	Biopsy positive group	Biopsy negative group	P
N	609	454	155	
age, year	68.32 ± 8.01	69.09 ± 8.06	66.07 ± 7.45	<0.01
BMI, kg/m²	24.22 (22.49–26.14)	24.22 (22.49–26.30)	24.24 (22.49–25.86)	0.482
TPSA, ng/ml	10.8 (87.36–17.45)	10.85 (7.26–17.19)	9.4 (6.22–14.04)	<0.001
transverse diameter, cm	4.9 (4.5–5.3)	4.8 (4.4–5.2)	5.2 (4.8–5.7)	<0.001
anteroposterior diameter, cm	3.4 (2.9–4.0)	3.3 (2.9–3.8)	3.9 (3.3–4.5)	<0.001
cephalocaudal diameter, cm	4.0 (3.5–4.7)	3.9 (3.4–4.425)	4.5 (3.8–5.2)	<0.001
Lesion’s longest diameter, cm	1.5 (1–2)	1.5 (1–2)	1.44 (1–1.97)	0.138
PI-RADS v2 score, number (percent)				
3	197 (32.35)	86 (18.94)	111 (71.61)	
4	241 (39.57)	204 (44.93)	37 (23.87)	<0.001
5	171 (27.97)	164 (36.12)	7 (4.52)	

#### Divide the Patient Evenly Into 5 Layers by DRS

The formula for calculating the disease risk score by the model is as follows:


$$DRS=\frac{1}{e^{-(-5.57+0.062X1+0.055X2+0.015X3-0.531X4-0.171X5-0.374X6-0.301X7+1.680X8)}}$$

X1 = AGE (year), X2 = BMI (kg/m²), X3 = TPSA (ng/ml), X4 = transverse diameter (cm), X5 = anteroposterior diameter (cm), X6 = cephalocaudal diameter (cm), X7 = Lesion’s longest diameter (cm), X8 = PI-RADS v2 score(number).

We included the following seven factors (X1-X7) because we conducted the paired chi-square test on these factors and found that these seven factors were meaningful to our study. The results are shown in the supplementary table (**Supplementary Tables 6–13**). Then the participation were equally divided into 5 layers according to the percentiles of their DRS, that is, Min–P20, P20–P40, P40–P60, P60–P80, and P80–Max.

#### Simulation and Model Establishment

The pathologic outcomes according to different sampling scheme from 1 core to 12 cores, sensitivities, NPV, accuracy and AUC with 95%CI are shown in **Table 2** (We only display the statistical results of the first layer, and the statistical results of the remaining 4 layers are displayed in the **Supplement Tables 1–4**). We analyzed AUC, when the lower boundary of the two-sided 95%CI was ≥95%, the number of cores is the most suitable. According to this standard, we chose 9 cores for layer 1, 6 cores for layer 2, 5 cores for layer 3, 4 cores for layer 4, and 4 cores for layer 5.At the same time, these selected number of cores also show a very high accuracy (layer 1: 99.18%; layer 2: 97.52%; layer 3: 97.56%; layer 4: 97.52%; layer 5: 95.90%). In addition, the first level missed 3 patients (3 csPCa), the second level missed 3 patients (3 csPCa), the third level missed 3 patients (2 csPCa), the fourth level missed 3 patients (3 csPCa), the fifth level missed 5 patients (5 csPCa). The data did not change significantly after including the concept of clinically significant prostate cancer.

**Table 2:** Detection rate of prostate cancer by different biopsy sampling schemes compared with that of 12-core systematic biopsy as the reference standard in layer 1.

The number of layers	CORE	12SBx (POSITIVE)		P (McNemar’s test)	Sensitivity (%)	NPV (%)	Accuracy (%)	AUC (95% CI)	P (AUC)
1	1	POSITIVE	17	0	48.57	82.86	85.25	0.743 (0.631–0.855)	<0.001
		NEGATIVE	18						
	2	POSITIVE	25	0	71.43	89.69	91.80	0.857 (0.765–0.95)	<0.001
		NEGATIVE	10						
	3	POSITIVE	NA	NA	NA	NA	NA	NA	NA
		NEGATIVE	NA						
	4	POSITIVE	27	0	77.14	91.58	93.44	0.886 (0.801–0.970)	<0.001
		NEGATIVE	8						
	5	POSITIVE	28	0.008	80.00	92.55	94.26	0.900 (0.820–0.980)	<0.001
		NEGATIVE	7						
	6	POSITIVE	30	0.063	85.71	94.57	95.90	0.929 (0.860–0.998)	<0.001
		NEGATIVE	5						
	7	POSITIVE	31	0.125	88.57	95.60	96.72	0.943 (0.880–1.000)	<0.001
		NEGATIVE	4						
	8	POSITIVE	NA	NA	NA	NA	NA	NA	NA
		NEGATIVE	NA						
	9	POSITIVE	34	1	97.14	98.86	99.18	0.986 (0.954–1.000)	<0.001
		NEGATIVE	1						
	10	POSITIVE	35	1	100	100	100	1.000 (1.000–1.000)	<0.001
		NEGATIVE	0						
	11	POSITIVE	35	1	100	100	100	1.000 (1.000–1.000)	<0.001
		NEGATIVE	0						

NA, NO ANSWER.

### Verification of the Model Using Data From the Fujian Union Hospital Cohort

A total of 279 patients with positive pathological results from the Fujian Union were included and stratified according to the method established by the Changhai cohort. Based on this principle, re-simulation was carried out for core reduction in patients in the Fujian Union cohort (**Table 3**). No patients were in the first layer. In the other layers, except for the second layer (88%), the accuracy rate were very considerable (The second-layer patients have a lower disease risk score than the other 3 layers, indicating that these patients have a relatively low risk of disease. More cores were needed to be taken for such patients, so the core reduction showed a relatively low accuracy, but the value of 88% we think is also ideal). However, the accuracy of each layer would be greatly improved, especially the third and fourth and even reaching 100%, when ignoring the non-clinically significant PCa.

**Table 3:** Analysis of External Validation Data of Fujian Union Hospital.

The number of layer	Core number	Total number	Number of positives after core reduction	Accuracy (ISUP ≥1)	Accuracy (ISUP ≥2)
2	6	25	22	88%	96.00%
3	5	28	27	96.43%	100%
4	4	78	74	94.87%	100%
5	4	148	140	94.59%	97.30%

### Establishment of the Website for Clinical Application

**Figure 5:**
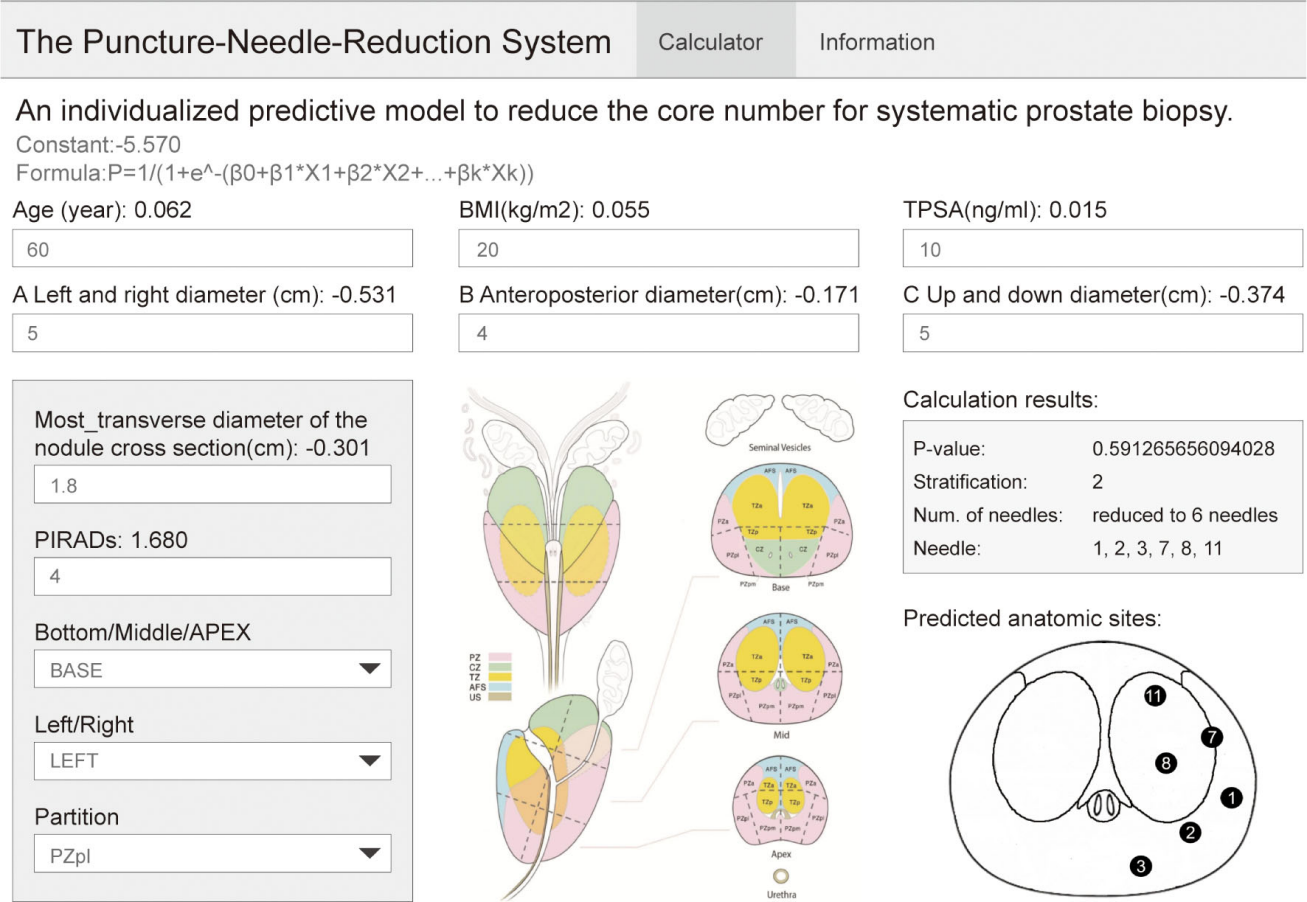
Website application display example.

As shown in **Figure 5**, the biopsy point distribution diagram of Changhai Hospital was adopted, and the relevant data of patients with PI-RADS score 3 and above reported by mpMRI was entered into the website. Among these, the lesion location website adopts the prostate division method used in PI-RADSV2.0 (18) and V2.1. The operator judges the possible positions of the lesion based on mpMRI. The position of the nodule location is divided into three areas: in the prostate BAES/MID/APEX, on the left or right side of the prostate, and the cross-sectional area of the prostate (divided according to PI-RADS V2.1). The website then outputs the DRS of the patient and the optimal number of cores, and our recommended biopsy point distribution diagram (The logic of website creation appendix in **Supplementary Table 5**).

## Discussion

The PRECISION study (6) showed that MRI-targeted biopsy could be minimally invasive, have few side effects, identify a high proportion of men who would benefit from treatment, and minimize the identification of men with clinically insignificant cancer in order to prevent overtreatment. The MRI-FIRST study (20) also pointed out that there is no significant difference between simple MRI-targeted biopsy and simple systematic biopsy for the detection of clinically significant prostate cancer (32.3% vs. 29.9%, p = 0.38); however, if the two methods are combined, the positive rate of clinically significant prostate cancer can reach 66%. In another multicenter prospective study 4M (21), the diagnostic efficacy of simple MRI-targeted biopsy and simple systematic biopsy for clinically significant prostate cancer was also compared; there was no significant difference between the two (23% vs. 25%, p = 0.17). However, systematic puncture can increase the positive rate of clinically insignificant prostate cancer (25% vs. 14%, p <0.0001).

Over the past few years many new technologies have become available for the management of Pca (22), but research on whether MRI-targeted biopsy can replace systematic biopsy has plateaued. Therefore, many compromised core reduction methods have also been studied and compared with similar studies; our research has two major advantages.

First, our research included as much mpMRI data as possible. In a recent Cochrane meta-analysis (23), mpMRI had a pooled sensitivity of 0.91 (95%CI: 0.83–0.95) and a pooled specificity of 0.37 (95%CI: 0.29–0.46) for ISUP grade >2 cancers. Several studies found that the PI-RADS score was a significant independent predictor of csPCa at biopsy (24, 25). In studies such as that of Hu et al. (16), this type of research did not include mpMRI data and only included tumor-related factors, and found that for patients with a PSA concentration of 20 ng/ml or higher, a 6-core systematic biopsy is preferred. However, we obtained fewer biopsy cores (5 cores and 4 cores) under stricter statistical conditions; therefore, we included the mpMRI data, especially the PI-RADS score, which is an important factor for core reduction. We included not only the scores for the lesions (PI-RADS score), but also the location of the lesions reported by mpMRI, which is a very important reason for the reduction in the number of cores.

Second, our research is the first to use modeling methods to combine basic patient information such as age, BMI, PSA, and indicators involved in mpMRI (transverse diameter, anteroposterior diameter, cephalocaudal diameter, lesion longest diameter, and PI-RADS v2 score). In a study by Shen (17), the biopsy point distribution diagram can be reduced to TBx + lateral 6-SBx based on TBx + 12-SBx, which means that the systematic biopsy core number can be reduced to 6 based on 12. The results of this study were also not satisfactory for the reduction of the core number (6 cores vs. 5/4 cores) because it did not achieve individualization; it did not include the age, PSA, BMI of the patient and other factors, or did not integrate these factors for individualized analysis. Age (26), BMI (27), PSA (28), three diameters of prostate, etc., have been confirmed as having a strong correlation factor with prostate cancer, and have their own significance in the impact on core number. However, if analyzed separately, they will often produce the opposite result. For example, if a patient has a higher PSA indicator (indicating fewer cores) but a smaller cancer lesion (indicating more puncture needles). However, studies that comprehensively consider these indicators are still insufficient.

This study is the first to introduce the concept of DRS value into model establishment for the comprehensive treatment of patients with a relatively comprehensive stratification of various factors. Through this processing method, more factors are integrated, and the layered processing makes the core number reduction more gradual, making it possible to reduce more biopsy cores, enabling us to achieve individualization in the true sense for the first time. Moreover, our study is also the first to consider the position of the lesion and perform personalized core reduction for each patient based on the position of the lesion through the website.

We successfully established an individualized model and established a website. Using this model, patients were equally divided into five layers. To ensure the detection rate, the number of cores can be reduced to less than 6 cores for more than half of the patients with lesions (PI-RADS ≥3 points). The website can directly provide the recommended number of systematic biopsy cores and recommended biopsy point distribution diagram, which is convenient for the promotion of the model among multiple centers and the development of follow-up prospective studies. After the patient is admitted to the hospital, the clinician will need to input patient information into the website, and then, with the assistance of the imaging doctor or the clinician’s own judgment of mpMRI, select the location of the lesion; the recommended core numbers and biopsy point distribution diagram will then be obtained. The surgeon can choose to use or not to use targeted biopsy according to the situation of the center and the patient.

This study has some limitations: First, the small sample size resulted in too few patients included in the first two layers in the external verification, which has a great impact on the result. Similarly, the inclusion of fewer patients in the first and second layers and more patients in the last three layers in the external verification reflects the imbalance of regional medical standards when China conducts multi-center research; screening levels in Shanghai and other regions are much higher than those in other regions (29), screening for prostate cancer remains inadequate in other regions. Second, the simulated process of reducing the number of cores is a human operation, and there is the influence of human subjective bias leading to instability of the model. Finally, this study adopts the method of grouping by DRS, resulting in a large difference in the number of pathologically negative and pathologically positive patients in each layer, especially the fifth layer. The statistical test efficiency will be affected by this, which requires us to perform follow-up prospective randomized research.

In conclusion, we are the first to propose a practical and feasible model of core reduction that considers the individual factors of each patient. Through the establishment of the website, the clinical application of the model becomes possible. For patients with suspicious lesions reported by mpMRI, we successfully reduced the number of cores to a minimum of 4.

## Data Availability Statement

The raw data supporting the conclusions of this article will be made available by the authors, without undue reservation.

## Ethics Statement

Ethical review and approval was not required for the study on human participants in accordance with the local legislation and institutional requirements. Written informed consent for participation was not required for this study in accordance with the national legislation and the institutional requirements.

## Author Contributions

XG, ZeC, MQ, ML, and CW contributed to conception and design of the study. ZeC, MQ, SJ, ZhC, and XS organized the database. CW, ZeC, SJ, and XS performed the statistical analysis. ZeC wrote the first draft of the manuscript. MQ, JJ, WZ, YW, SJ, JZ, and LL wrote sections of the manuscript. WZ and JJ established the website. All authors contributed to the article and approved the submitted version.

## Funding

The study was supported by the Promote Clinical Skills and Innovation Ability of Municipal Hospitals Project (Grant number: SHDC2020CR6007).

## Conflict of Interest

The authors declare that the research was conducted in the absence of any commercial or financial relationships that could be construed as a potential conflict of interest.

## Publisher’s Note

All claims expressed in this article are solely those of the authors and do not necessarily represent those of their affiliated organizations, or those of the publisher, the editors and the reviewers. Any product that may be evaluated in this article, or claim that may be made by its manufacturer, is not guaranteed or endorsed by the publisher.

## Abbreviations

TPSA, total prostate-specific antigen; 12-SBx,12-core systematic biopsy; TBx, targeted biopsy; csPCa, clinically significant prostate cancer; mpMRI, multiparametric magnetic resonance imaging; TRUS, transrectal ultrasound guided; AUC, Area Under the ROC Curve; BMI, Body Mass Index; PCa, Prostate Cancer; PSA, Prostate Specific Antigen; DRS, Disease Risk Score; CI, confidence intervals; NPV, negative prediction value.

## References

[1] Hodge KK McNeal JE Terris MK Stamey TA. Random Systematic Versus Directed Ultrasound Guided Transrectal Core Biopsies of the Prostate. J Urol (1989) 142:71–4. doi: 10.1016/S0022-5347(17)38664-0 2659827

[2] Babaian RJ Toi A Kamoi K Troncoso P Sweet J Evans R. A Comparative Analysis of Sextant and an Extended 11-Core Multisite Directed Biopsy Strategy. J Urol (2000) 163:152–7. doi: 10.1016/S0022-5347(05)67993-1 10604335

[3] Seles M Gutschi T Mayrhofer K Fischereder K Ehrlich G Galle G. Sampling of the Anterior Apical Region Results in Increased Cancer Detection and Upgrading in Transrectal Repeat Saturation Biopsy of the Prostate. BJU Int (2016) 117:592–7. doi: 10.1111/bju.13108 25726856

[4] Smeenge M de la Rosette J Wijkstra H. Current Status of Transrectal Ultrasound Techniques in Prostate Cancer. Curr Opin Urol (2012) 22:297–302. doi: 10.1097/MOU.0b013e3283548154 22595778

[5] D’Amico A Tempany C Cormack R Hata N Jinzaki M Tuncali K. Transperineal Magnetic Resonance Image Guided Prostate Biopsy. AUA J (2000) 164:385–7. doi: 10.1016/S0022-5347(05)67366-1 10893591

[6] Kasivisvanathan V Rannikko AS Borghi M Panebianco V Mynderse LA Vaarala MH. MRI-Targeted or Standard Biopsy for Prostate-Cancer Diagnosis. N Engl J Med (2018) 378:1767–77. doi: 10.1056/NEJMoa1801993 PMC9084630 29552975

[7] Baco E Rud E Eri LM Moen G Vlatkovic L Svindland A. A Randomized Controlled Trial To Assess and Compare the Outcomes of Two-Core Prostate Biopsy Guided by Fused Magnetic Resonance and Transrectal Ultrasound Images and Traditional 12-Core Systematic Biopsy. Eur Urol (2016) 69:149–56. doi: 10.1016/j.eururo.2015.03.041 25862143

[8] Siddiqui MM Rais-Bahrami S Turkbey B George AK Rothwax J Shakir N. Comparison of MR/ultrasound Fusion-Guided Biopsy With Ultrasound-Guided Biopsy for the Diagnosis of Prostate Cancer. JAMA (2015) 313:390–7. doi: 10.1001/jama.2014.17942 PMC4572575 25626035

[9] Richenberg J Logager V Panebianco V Rouviere O Villeirs G Schoots IG. The Primacy of Multiparametric MRI in Men With Suspected Prostate Cancer. Eur Radiol (2019) 29:6940–52. doi: 10.1007/s00330-019-06166-z PMC6828624 31172275

[10] Sonn GA Fan RE Ghanouni P Wang NN Brooks JD Loening AM. Prostate Magnetic Resonance Imaging Interpretation Varies Substantially Across Radiologists. Eur Urol Focus (2019) 5:592–9. doi: 10.1016/j.euf.2017.11.010 29226826

[11] Lu AJ Syed JS Ghabili K Hsiang WR Nguyen KA Leapman MS. Role of Core Number and Location in Targeted Magnetic Resonance Imaging-Ultrasound Fusion Prostate Biopsy. Eur Urol (2019) 76:14–7. doi: 10.1016/j.eururo.2019.04.008 31047733

[12] Tu X Lin T Cai D Liu Z Yang L Wei Q. The Optimal Core Number and Site for MRI-Targeted Biopsy of Prostate? A Systematic Review and Pooled Analysis. Minerva Urol e Nefrologica = Ital J Urol Nephrol (2020) 72:144–51. doi: 10.23736/S0393-2249.20.03639-5 32003207

[13] Sathianathen N Omer A Harriss E Davies L Kasivisvanathan V Punwani S. Negative Predictive Value of Multiparametric Magnetic Resonance Imaging in the Detection of Clinically Significant Prostate Cancer in the Prostate Imaging Reporting and Data System Era: A Systematic Review and Meta-Analysis. Europ Urol (2020) 78:402–14. doi: 10.1016/j.eururo.2020.03.048 32444265

[14] Cornford P van den Bergh RCN Briers E Van den Broeck T Cumberbatch MG De Santis M. EAU-EANM-ESTRO-ESUR-SIOG Guidelines on Prostate Cancer. Part II—2020 Update: Treatment of Relapsing and Metastatic Prostate Cancer. Eur Urol (2021) 79:263–82. doi: 10.1016/j.eururo.2020.09.046 33039206

[15] Merriel SWD Funston G Hamilton W. Prostate Cancer in Primary Care. Adv Ther (2018) 35:1285–94. doi: 10.1007/s12325-018-0766-1 PMC6133140 30097885

[16] Hu Z Wang J Sun D Cui L Ran W. How Many Cores Does Systematic Prostate Biopsy Need?: A Large-Sample Retrospective Analysis. J Ultrasound Med (2019) 38:1491–9. doi: 10.1002/jum.14834 30380169

[17] Shen WW Cui LG Ran WQ Sun Y Jiang J Pei XL. Targeted Biopsy With Reduced Number of Cores: Optimal Sampling Scheme in Patients Undergoing Magnetic Resonance Imaging/Transrectal Ultrasound Fusion Prostate Biopsy. Ultrasound Med Biol (2020) 46:1197–207. doi: 10.1016/j.ultrasmedbio.2020.01.017 32107089

[18] Weinreb JC Barentsz JO Choyke PL Cornud F Haider MA Macura KJ. PI-RADS Prostate Imaging - Reporting and Data System: 2015, Version 2. Eur Urol (2016) 69:16–40. doi: 10.1016/j.eururo.2015.08.052 26427566 PMC6467207

[19] Wang H Lin H He B Guo X Zhou Y Xi P. A Novel Perineal Nerve Block Approach for Transperineal Prostate Biopsy: An Anatomical Analysis-Based Randomized Single-Blind Controlled Trial. Urology (2020) 146:25–31. doi: 10.1016/j.urology.2020.01.058 32335086

[20] Rouvière O Puech P Renard-Penna R Claudon M Roy C Mège-Lechevallier F. Use of Prostate Systematic and Targeted Biopsy on the Basis of Multiparametric MRI in Biopsy-Naive Patients (MRI-FIRST): A Prospective, Multicentre, Paired Diagnostic Study. Lancet Oncol (2019) 20:100–9. doi: 10.1016/s1470-2045(18)30569-2 30470502

[21] van der Leest M Cornel E Israël B Hendriks R Padhani A Hoogenboom M. Head-To-Head Comparison of Transrectal Ultrasound-Guided Prostate Biopsy Versus Multiparametric Prostate Resonance Imaging With Subsequent Magnetic Resonance-Guided Biopsy in Biopsy-Naïve Men With Elevated Prostate-Specific Antigen: A Large Prospective Multicenter Clinical Study. European Urology (2019) 75:570–8. doi: 10.1016/j.eururo.2018.11.023 30477981

[22] Checcucci E Amparore D De Luca S Autorino R Fiori C Porpiglia F. Precision Prostate Cancer Surgery: An Overview of New Technologies and Techniques. Minerva Urol e Nefrologica = Ital J Urol Nephrol (2019) 71:487–501. doi: 10.23736/S0393-2249.19.03365-4 30700084

[23] Drost FH Osses DF Nieboer D Steyerberg EW Bangma CH Roobol MJ. Prostate MRI, With or Without MRI-Targeted Biopsy, and Systematic Biopsy for Detecting Prostate Cancer. Cochrane Database Syst Rev (2019) 4:CD012663. doi: 10.1002/14651858.CD012663.pub2 31022301 PMC6483565

[24] Distler FA Radtke JP Bonekamp D Kesch C Schlemmer HP Wieczorek K. The Value of PSA Density in Combination With PI-RADS™ for the Accuracy of Prostate Cancer Prediction. J Urol (2017) 198:575–82. doi: 10.1016/j.juro.2017.03.130 28373135

[25] Washino S Okochi T Saito K Konishi T Hirai M Kobayashi Y. Combination of Prostate Imaging Reporting and Data System (PI-RADS) Score and Prostate-Specific Antigen (PSA) Density Predicts Biopsy Outcome in Prostate Biopsy Naïve Patients. BJU Int (2017) 119:225–33. doi: 10.1111/bju.13465 26935594

[26] Vinjamoori AH Jagannathan JP Shinagare AB Taplin ME Oh WK Van den Abbeele AD. Atypical Metastases From Prostate Cancer: 10-Year Experience at a Single Institution. AJR. Am J Roentgenol (2012) 199:367–72. doi: 10.2214/AJR.11.7533 22826398

[27] He B Chen R Gao X Ren S Yang B Hou J. Nomograms for Predicting Gleason Upgrading in a Contemporary Chinese Cohort Receiving Radical Prostatectomy After Extended Prostate Biopsy: Development and Internal Validation. Oncotarget (2016) 7:17275–85. doi: 10.18632/oncotarget.7787 PMC4941387 26943768

[28] Welch H Albertsen P. Reconsidering Prostate Cancer Mortality - The Future of PSA Screening. N Engl J Med (2020) 382:1557–63. doi: 10.1056/NEJMms1914228 32294352

[29] Fu Z Guo X Zhang S Zheng R Zeng H Chen R. Statistical Analysis of Incidence and Mortality of Prostate Cancer in China, 2015. Chinese J Onco (2020) 42:718–22. doi: 10.3760/cma.j.cn112152-20200313-00200 32988152

