# Supplementary material for: Establishment of an Individualized Predictive Model to Reduce the Core Number for Systematic Prostate Biopsy: A Dual Center Study Based on Stratification of the Disease Risk Score
Source: Front Oncol. 2022 Feb 14;11:831603. doi: 10.3389/fonc.2021.831603 (PMC8882832; doi:10.3389/fonc.2021.831603)
Supplement: Supplementary file 1 [file Table_1.docx]

Table 1 Detection rate of prostate cancer by different biopsy sampling schemes compared with that of 12-core systematic biopsy as the reference standard in layer 2.

| The number of layers | CORE | 12SBx（POSITIVE） | | P（McNemar’s test） | Sensitivity  (%) | NPV  (%) | Accuracy  (%) | AUC (95% CI) | P （AUC） |
| --- | --- | --- | --- | --- | --- | --- | --- | --- | --- |
| 2 | 1 | POSITIVE | 53 | 0 | 63.86 | 55.88 | 75.21 | 0.819（0.747-0.892） | <0.001 |
|  |  | NEGATIVE | 30 |  |  |  |  |  |  |
|  | 2 | POSITIVE | 66 | 0 | 79.52 | 69.09 | 85.95 | 0.898（0.842-0.953） | <0.001 |
|  |  | NEGATIVE | 27 |  |  |  |  |  |  |
|  | 3 | POSITIVE | 73 | 0.002 | 87.95 | 79.17 | 91.74 | 0.940（0.897-0.983） | <0.001 |
|  |  | NEGATIVE | 10 |  |  |  |  |  |  |
|  | 4 | POSITIVE | 76 | 0.016 | 91.57 | 84.44 | 94.21 | 0.958(0.922-0.994) | <0.001 |
|  |  | NEGATIVE | 7 |  |  |  |  |  |  |
|  | 5 | POSITIVE | 79 | 0.125 | 95.18 | 90.48 | 96.69 | 0.976(0.949-1.000) | <0.001 |
|  |  | NEGATIVE | 4 |  |  |  |  |  |  |
|  | 6 | POSITIVE | 80 | 0.25 | 96.39 | 92.68 | 97.52 | 0.982(0.980-1.000) | <0.001 |
|  |  | NEGATIVE | 3 |  |  |  |  |  |  |
|  | 7 | POSITIVE | NA | NA | NA | NA | NA | NA | NA |
|  |  | NEGATIVE | NA |  |  |  |  |  |  |
|  | 8 | POSITIVE | NA | NA | NA | NA | NA | NA | NA |
|  |  | NEGATIVE | NA |  |  |  |  |  |  |
|  | 9 | POSITIVE | 82 | 1 | 98.80 | 97.44 | 99.17 | 0.994(0.980-1.000) | <0.001 |
|  |  | NEGATIVE | 1 |  |  |  |  |  |  |
|  | 10 | POSITIVE | NA | NA | NA | NA | NA | NA | NA |
|  |  | NEGATIVE | NA |  |  |  |  |  |  |
|  | 11 | POSITIVE | 83 | 1 | 100 | 100 | 100 | 1.000(1.000-1.000) | <0.001 |
|  |  | NEGATIVE | 0 |  |  |  |  |  |  |

Table 2 Detection rate of prostate cancer by different biopsy sampling schemes compared with that of 12-core systematic biopsy as the reference standard in layer 3.

| The number of layers | CORE | 12SBx（POSITIVE） | | P（McNemar’s test） | Sensitivity  (%) | NPV  (%) | Accuracy  (%) | AUC (95% CI) | P （AUC） |
| --- | --- | --- | --- | --- | --- | --- | --- | --- | --- |
| 3 | 1 | POSITIVE | 72 | 0 | 66.67 | 29.41 | 70.73 | 0.833（0.760-0.907） | <0.001 |
|  |  | NEGATIVE | 36 |  |  |  |  |  |  |
|  | 2 | POSITIVE | 86 | 0 | 79.63 | 40.54 | 82.11 | 0.898（0.843-0.953） | <0.001 |
|  |  | NEGATIVE | 22 |  |  |  |  |  |  |
|  | 3 | POSITIVE | 96 | 0 | 88.89 | 55.56 | 90.24 | 0.944（0.906-0.983） | NA |
|  |  | NEGATIVE | 12 |  |  |  |  |  |  |
|  | 4 | POSITIVE | 101 | 0.016 | 93.52 | 68.18 | 94.31 | 0.968(0.939-0.997) | <0.001 |
|  |  | NEGATIVE | 7 |  |  |  |  |  |  |
|  | 5 | POSITIVE | 105 | 0. 25 | 97.22 | 83.33 | 97.56 | 0.986(0.967-1.000) | <0.001 |
|  |  | NEGATIVE | 3 |  |  |  |  |  |  |
|  | 6 | POSITIVE | 107 | 1 | 99.07 | 93.75 | 99.19 | 0.995(0.985-1.000) | <0.001 |
|  |  | NEGATIVE | 1 |  |  |  |  |  |  |
|  | 7 | POSITIVE | NA | NA | NA | NA | NA | NA | NA |
|  |  | NEGATIVE | NA |  |  |  |  |  |  |
|  | 8 | POSITIVE | NA | NA | NA | NA | NA | NA | NA |
|  |  | NEGATIVE | NA |  |  |  |  |  |  |
|  | 9 | POSITIVE | 108 | 1 | 100 | 100 | 100 | 1.000(1.000-1.000) | <0.001 |
|  |  | NEGATIVE | 0 |  |  |  |  |  |  |
|  | 10 | POSITIVE | 108 | 1 | 100 | 100 | 100 | 1.000(1.000-1.000) | <0.001 |
|  |  | NEGATIVE | 0 |  |  |  |  |  |  |
|  | 11 | POSITIVE | 108 | 1 | 100 | 100 | 100 | 1.000(1.000-1.000) | <0.001 |
|  |  | NEGATIVE | 0 |  |  |  |  |  |  |

Table 3 Detection rate of prostate cancer by different biopsy sampling schemes compared with that of 12-core systematic biopsy as the reference standard in layer 4.

| The number of layers | CORE | 12SBx（POSITIVE） | | P（McNemar’s test） | Sensitivity  (%) | NPV  (%) | Accuracy  (%) | AUC (95% CI) | P （AUC） |
| --- | --- | --- | --- | --- | --- | --- | --- | --- | --- |
| 4 | 1 | POSITIVE | 81 | 0 | 75.00 | 32.50 | 77.69 | 0.875（0.811-0.939） | <0.001 |
|  |  | NEGATIVE | 27 |  |  |  |  |  |  |
|  | 2 | POSITIVE | 90 | 0 | 83.33 | 41.94 | 85.12 | 0.917（0.867-0.966） | <0.001 |
|  |  | NEGATIVE | 18 |  |  |  |  |  |  |
|  | 3 | POSITIVE | 100 | 0.008 | 92.59 | 61.90 | 93.39 | 0.963（0.931-0.995） | <0.001 |
|  |  | NEGATIVE | 8 |  |  |  |  |  |  |
|  | 4 | POSITIVE | 105 | 0.25 | 97.22 | 81.25 | 97.52 | 0.986(0.967-1.000) | <0.001 |
|  |  | NEGATIVE | 3 |  |  |  |  |  |  |
|  | 5 | POSITIVE | 107 | 1 | 99.07 | 92.86 | 99.17 | 0.995(0.985-1.000) | <0.001 |
|  |  | NEGATIVE | 1 |  |  |  |  |  |  |
|  | 6 | POSITIVE | 108 | 1 | 100 | 100 | 100 | 1.000(1.000-1.000) | <0.001 |
|  |  | NEGATIVE | 0 |  |  |  |  |  |  |
|  | 7 | POSITIVE | 108 | 1 | 100 | 100 | 100 | 1.000(1.000-1.000) | <0.001 |
|  |  | NEGATIVE | 0 |  |  |  |  |  |  |
|  | 8 | POSITIVE | 108 | 1 | 100 | 100 | 100 | 1.000(1.000-1.000) | <0.001 |
|  |  | NEGATIVE | 0 |  |  |  |  |  |  |
|  | 9 | POSITIVE | 108 | 1 | 100 | 100 | 100 | 1.000(1.000-1.000) | <0.001 |
|  |  | NEGATIVE | 0 |  |  |  |  |  |  |
|  | 10 | POSITIVE | 108 | 1 | 100 | 100 | 100 | 1.000(1.000-1.000) | <0.001 |
|  |  | NEGATIVE | 0 |  |  |  |  |  |  |
|  | 11 | POSITIVE | 108 | 1 | 100 | 100 | 100 | 1.000(1.000-1.000) | <0.001 |
|  |  | NEGATIVE | 0 |  |  |  |  |  |  |

Table 4 Detection rate of prostate cancer by different biopsy sampling schemes compared with that of 12-core systematic biopsy as the reference standard in layer 5.

| The number of layers | CORE | 12SBx（POSITIVE） | | P（McNemar’s test） | Sensitivity  (%) | NPV  (%) | Accuracy  (%) | AUC (95% CI) | P （AUC） |
| --- | --- | --- | --- | --- | --- | --- | --- | --- | --- |
| 5 | 1 | POSITIVE | 95 | 0 | 79.17 | 7.41 | 79.51 | 0.896（0.799-0.992） | <0.001 |
|  |  | NEGATIVE | 25 |  |  |  |  |  |  |
|  | 2 | POSITIVE | 107 | 0 | 89.17 | 13.33 | 89.34 | 0.946（0.889-1.000） | <0.001 |
|  |  | NEGATIVE | 13 |  |  |  |  |  |  |
|  | 3 | POSITIVE | 114 | 0.031 | 95.00 | 25.00 | 95.08 | 0.975（0.943-1.000） | <0.001 |
|  |  | NEGATIVE | 6 |  |  |  |  |  |  |
|  | 4 | POSITIVE | 115 | 0.063 | 95.83 | 28.57 | 95.90 | 0.979(0.951-1.000) | <0.001 |
|  |  | NEGATIVE | 5 |  |  |  |  |  |  |
|  | 5 | POSITIVE | 119 | 1 | 99.17 | 66.67 | 99.18 | 0.996(0.985-1.000) | <0.001 |
|  |  | NEGATIVE | 1 |  |  |  |  |  |  |
|  | 6 | POSITIVE | 120 | 1 | 100 | 100 | 100 | 1.000(1.000-1.000) | <0.001 |
|  |  | NEGATIVE | 0 |  |  |  |  |  |  |
|  | 7 | POSITIVE | 120 | 1 | 100 | 100 | 100 | 1.000(1.000-1.000) | <0.001 |
|  |  | NEGATIVE | 0 |  |  |  |  |  |  |
|  | 8 | POSITIVE | 120 | 1 | 100 | 100 | 100 | 1.000(1.000-1.000) | <0.001 |
|  |  | NEGATIVE | 0 |  |  |  |  |  |  |
|  | 9 | POSITIVE | 120 | 1 | 100 | 100 | 100 | 1.000(1.000-1.000) | <0.001 |
|  |  | NEGATIVE | 0 |  |  |  |  |  |  |
|  | 10 | POSITIVE | 120 | 1 | 100 | 100 | 100 | 1.000(1.000-1.000) | <0.001 |
|  |  | NEGATIVE | 0 |  |  |  |  |  |  |
|  | 11 | POSITIVE | 120 | 1 | 100 | 100 | 100 | 1.000(1.000-1.000) | <0.001 |
|  |  | NEGATIVE | 0 |  |  |  |  |  |  |

Table 5 The logic of website creation.

| BAES/MID/APEX | L/R | ZONE | 3CORE | 4 CORE | 5 CORE | 6 CORE | 9 CORE |
| --- | --- | --- | --- | --- | --- | --- | --- |
| BASE | LIFT | PZpl | 1 2 7 | 1 2 3 7 | 1 2 3 7 8 | 1 2 3 7 8 11 | 1 2 3 4 5 7 8 9 11 |
|  |  | PZa | 1 7 8 | 1 2 7 8 | 1 2 3 7 8 | 1 2 3 7 8 11 | 1 2 3 4 7 8 9 11 12 |
|  |  | CZ | 1 2 7 | 1 2 3 4 | 1 2 3 4 8 | 1 2 3 4 7 8 | 1 2 3 4 5 7 8 9 11 |
|  |  | TZp | 3 7 8 | 3 7 8 11 | 3 7 8 9 11 | 2 3 7 8 9 11 | 1 2 3 4 5 7 8 9 11 |
|  |  | TZa | 7 11 12 | 7 8 11 12 | 2 7 8 11 12 | 1 2 7 8 11 12 | 1 2 3 4 7 8 9 11 12 |
|  |  | AFS | 8 11 12 | 7 8 11 12 | 7 8 9 11 12 | 7 8 9 10 11 12 | 1 2 3 4 7 8 9 11 12 |
|  | RIGHT | PZpl | 5 6 10 | 4 5 6 10 | 4 5 6 9 10 | 4 5 6 9 10 12 | 2 3 4 5 6 8 9 10  12 |
|  |  | PZa | 6 9 10 | 6 9 10 12 | 5 6 9 10 12 | 4 5 6 9 10 12 | 3 4 5 6 8 9 10 11 12 |
|  |  | CZ | 5 9 10 | 4 5 9 10 | 3 4 5 9 10 | 3 4 5 6 9 10 | 2 3 4 5 6 8 9 10  12 |
|  |  | TZp | 4 9 10 | 4 5 9 10 | 4 5 9 10 12 | 4 5 8 9 10 12 | 2 3 4 5 6 8 9 10  12 |
|  |  | TZa | 9 11 12 | 9 10 11 12 | 8 9 10 11 12 | 5 8 9 10 11 12 | 3 4 5 6 8 9 10 11 12 |
|  |  | AFS | 9 11 12 | 9 10 11 12 | 8 9 10 11 12 | 7 8 9 10 11 12 | 4 5 6 7 8 9 10 11 12 |
| MID | LIFT | PZpl | 1 2 7 | 1 2 3 7 | 1 2 3 7 8 | 1 2 3 7 8 11 | 1 2 3 4 5 7 8 9 11 |
|  |  | PZa | 1 7 8 | 1 2 7 8 | 1 2 3 7 8 | 1 2 3 7 8 11 | 1 2 3 4 7 8 9 11 12 |
|  |  | PZpm | 1 2 7 | 1 2 3 4 | 1 2 3 4 8 | 1 2 3 4 7 8 | 1 2 3 4 5 7 8 9 11 |
|  |  | TZp | 3 7 8 | 3 7 8 11 | 3 7 8 9 11 | 2 3 7 8 9 11 | 1 2 3 4 5 7 8 9 11 |
|  |  | TZa | 7 11 12 | 7 8 11 12 | 2 7 8 11 12 | 1 2 7 8 11 12 | 1 2 3 4 7 8 9 11 12 |
|  |  | AFS | 8 11 12 | 7 8 11 12 | 7 8 9 11 12 | 7 8 9 10 11 12 | 1 2 3 4 7 8 9 11 12 |
|  | RIGHT | PZpl | 5 6 10 | 4 5 6 10 | 4 5 6 9 10 | 4 5 6 9 10 12 | 2 3 4 5 6 8 9 10  12 |
|  |  | PZa | 6 9 10 | 6 9 10 12 | 5 6 9 10 12 | 4 5 6 9 10 12 | 3 4 5 6 8 9 10 11 12 |
|  |  | PZpm | 5 9 10 | 4 5 9 10 | 3 4 5 9 10 | 3 4 5 6 9 10 | 2 3 4 5 6 8 9 10  12 |
|  |  | TZp | 4 9 10 | 4 5 9 10 | 4 5 9 10 12 | 4 5 8 9 10 12 | 2 3 4 5 6 8 9 10  12 |
|  |  | TZa | 9 11 12 | 9 10 11 12 | 8 9 10 11 12 | 5 8 9 10 11 12 | 3 4 5 6 8 9 10 11 12 |
|  |  | AFS | 9 11 12 | 9 10 11 12 | 8 9 10 11 12 | 7 8 9 10 11 12 | 3 4 5 6 8 9 10 11 12 |
| APEX | LIFT | PZpl | 1 2 7 | 1 2 3 7 | 1 2 3 7 8 | 1 2 3 7 8 11 | 1 2 3 4 5 7 8 9 11 |
|  |  | PZa | 1 7 8 | 1 2 7 8 | 1 2 3 7 8 | 1 2 3 7 8 11 | 1 2 3 4 7 8 9 11 12 |
|  |  | PZpm | 1 2 7 | 1 2 3 4 | 1 2 3 4 8 | 1 2 3 4 7 8 | 1 2 3 4 5 7 8 9 11 |
|  |  | TZp | 3 7 8 | 3 7 8 11 | 3 7 8 9 11 | 2 3 7 8 9 11 | 1 2 3 4 5 7 8 9 11 |
|  |  | TZa | 7 11 12 | 7 8 11 12 | 2 7 8 11 12 | 1 2 7 8 11 12 | 1 2 3 4 7 8 9 11 12 |
|  |  | AFS | 8 11 12 | 7 8 11 12 | 7 8 9 11 12 | 7 8 9 10 11 12 | 1 2 3 4 7 8 9 11 12 |
|  | RIGHT | PZpl | 5 6 10 | 4 5 6 10 | 4 5 6 9 10 | 4 5 6 9 10 12 | 2 3 4 5 6 8 9 10  12 |
|  |  | PZa | 6 9 10 | 6 9 10 12 | 5 6 9 10 12 | 4 5 6 9 10 12 | 3 4 5 6 8 9 10 11 12 |
|  |  | PZpm | 5 9 10 | 4 5 9 10 | 3 4 5 9 10 | 3 4 5 6 9 10 | 2 3 4 5 6 8 9 10  12 |
|  |  | TZp | 4 9 10 | 4 5 9 10 | 4 5 9 10 12 | 4 5 8 9 10 12 | 2 3 4 5 6 8 9 10  12 |
|  |  | TZa | 9 11 12 | 9 10 11 12 | 8 9 10 11 12 | 5 8 9 10 11 12 | 3 4 5 6 8 9 10 11 12 |
|  |  | AFS | 9 11 12 | 9 10 11 12 | 8 9 10 11 12 | 7 8 9 10 11 12 | 3 4 5 6 8 9 10 11 12 |

Table 6-12: Show the results of the paired chi-square test for eachle factor

Table 6: the results of the paired chi-square test for age(year)

|  | AGE＞70 |  |  |  | AGE≤70 |  |  |
| --- | --- | --- | --- | --- | --- | --- | --- |
| core | positive | negative | Pvalue | core | positive | negative | Pvalue |
| 1 | 152 | 101 |  | 1 | 166 | 180 |  |
| 2 | 179 | 74 |  | 2 | 195 | 151 |  |
| 3 | 195 | 68 |  | 3 | 213 | 133 |  |
| 4 | 201 | 62 |  | 4 | 223 | 123 |  |
| 5 | 207 | 56 | 0.016 | 5 | 231 | 115 | 0.004 |
| 6 | 208 | 55 | 0.031 | 6 | 236 | 110 | 0.125 |
| 8 | 209 | 54 | 0.063 | 7 | 237 | 109 | 0.25 |
| 9 | 213 | 50 | 1 | 9 | 239 | 107 | 1 |
| 11 | 214 | 49 |  | 10 | 240 | 106 |  |

Table 7: the results of the paired chi-square test for BMI(kg/m2)

|  | BMI＜25 |  |  |  | BMI≥25 |  |  |
| --- | --- | --- | --- | --- | --- | --- | --- |
| core | positive | negative | Pvalue | core | positive | negative | Pvalue |
| 1 | 188 | 176 |  | 1 | 130 | 115 |  |
| 2 | 218 | 146 |  | 2 | 156 | 89 |  |
| 3 | 237 | 127 |  | 3 | 171 | 74 |  |
| 4 | 245 | 119 |  | 4 | 179 | 66 | 0 |
| 5 | 253 | 111 |  | 5 | 185 | 60 | 0.031 |
| 6 | 256 | 108 |  | 6 | 188 | 57 | 0.25 |
| 7 | 257 | 107 | 0.031 | 8 | 189 | 56 | 0.5 |
| 9 | 261 | 103 | 0.5 | 9 | 191 | 54 |  |
| 10 | 262 | 102 | 1 |  |  |  |  |
| 11 | 263 | 101 |  |  |  |  |  |

Table 8: the results of the paired chi-square test for transverse diameter (cm)

|  | transverse diameter＜4.9 |  |  |  | transverse diameter≥4.9 |  |  |
| --- | --- | --- | --- | --- | --- | --- | --- |
| core | positive | negative | Pvalue | core | positive | negative | Pvalue |
| 1 | 180 | 108 |  | 1 | 138 | 183 |  |
| 2 | 208 | 80 |  | 2 | 166 | 155 |  |
| 3 | 229 | 59 |  | 3 | 179 | 142 |  |
| 4 | 237 | 51 | 0.004 | 4 | 187 | 134 |  |
| 5 | 241 | 47 | 0.063 | 5 | 197 | 124 |  |
| 6 | 243 | 45 | 0.25 | 6 | 201 | 120 | 0.016 |
| 8 | 244 | 44 | 0.5 | 7 | 202 | 119 | 0.031 |
| 9 | 246 | 42 | 1 | 9 | 206 | 115 | 0.5 |
|  |  |  |  | 10 | 207 | 114 | 1 |
|  |  |  |  | 11 | 208 | 113 |  |

Table 9: the results of the paired chi-square test for anteroposterior diameter (cm)

|  | anteroposterior diameter≥3.4 |  |  |  | anteroposterior diameter＜3.4 |  |  |
| --- | --- | --- | --- | --- | --- | --- | --- |
| core | positive | negative | Pvalue | core | positive | negative | Pvalue |
| 1 | 149 | 187 |  | 1 | 169 | 104 |  |
| 2 | 179 | 157 |  | 2 | 195 | 78 |  |
| 3 | 197 | 139 |  | 3 | 211 | 62 |  |
| 4 | 204 | 132 |  | 4 | 220 | 53 | 0.001 |
| 5 | 211 | 125 |  | 5 | 227 | 46 | 0.063 |
| 6 | 215 | 121 | 0.016 | 6 | 229 | 44 | 0.25 |
| 7 | 216 | 120 | 0.031 | 8 | 230 | 43 | 0.5 |
| 9 | 220 | 116 | 0.5 | 9 | 232 | 41 | 1 |
| 10 | 221 | 115 | 1 |  |  |  |  |
| 11 | 222 | 114 |  |  |  |  |  |

Table 10: the results of the paired chi-square test for cephalocaudal diameter (cm)

|  | cephalocaudal diameter＜4 |  |  |  | cephalocaudal diameter≥4 |  |  |
| --- | --- | --- | --- | --- | --- | --- | --- |
| core | positive | negative | Pvalue | core | positive | negative | Pvalue |
| 1 | 169 | 118 |  | 1 | 149 | 166 |  |
| 2 | 204 | 83 |  | 2 | 170 | 145 |  |
| 3 | 220 | 67 |  | 3 | 188 | 127 |  |
| 4 | 231 | 56 | 0.002 | 4 | 193 | 122 |  |
| 5 | 237 | 50 | 0.125 | 5 | 194 | 121 |  |
| 6 | 238 | 49 | 0.25 | 6 | 199 | 116 | 0.016 |
| 8 | 239 | 48 | 0.5 | 7 | 200 | 115 | 0.031 |
| 9 | 241 | 46 | 1 | 9 | 204 | 111 | 0.5 |
|  |  |  |  | 10 | 205 | 110 | 1 |
|  |  |  |  | 11 | 206 | 109 |  |

Table 11: the results of the paired chi-square test for Lesion’s longest diameter (cm)

|  | Lesion’s longest diameter＜1.5 |  |  |  | Lesion’s longest diameter≥1.5 |  |  |
| --- | --- | --- | --- | --- | --- | --- | --- |
| core | positive | negative | Pvalue | core | positive | negative | Pvalue |
| 1 | 135 | 169 |  | 1 | 183 | 122 |  |
| 2 | 165 | 139 |  | 2 | 209 | 96 |  |
| 3 | 193 | 111 |  | 3 | 215 | 90 |  |
| 4 | 204 | 100 |  | 4 | 220 | 85 | 0.002 |
| 5 | 213 | 91 | 0.001 | 5 | 225 | 80 | 0.063 |
| 6 | 218 | 86 | 0.031 | 6 | 226 | 79 | 0.125 |
| 7 | 219 | 85 | 0.063 | 8 | 227 | 78 | 0.25 |
| 9 | 224 | 80 | 1 | 9 | 228 | 77 | 0.5 |
|  |  |  |  | 10 | 229 | 76 | 1 |
|  |  |  |  | 11 | 230 | 75 |  |

Table 12: the results of the paired chi-square test for PSA (ng/ml)

|  | PSA≥20 |  |  |  | PSA＜20 |  |  |
| --- | --- | --- | --- | --- | --- | --- | --- |
| core | positive | negative | Pvalue | core | positive | negative | Pvalue |
| 1 | 87 | 33 | 0 | 1 | 231 | 258 |  |
| 2 | 96 | 24 | 0.063 | 2 | 278 | 211 |  |
| 3 | 99 | 21 | 0.5 | 3 | 309 | 180 |  |
| 4 | 100 | 20 | 1 | 4 | 324 | 165 |  |
| 6 | 101 | 19 |  | 5 | 338 | 151 |  |
|  |  |  |  | 6 | 343 | 146 |  |
|  |  |  |  | 7 | 344 | 145 |  |
|  |  |  |  | 8 | 345 | 144 | 0.008 |
|  |  |  |  | 9 | 351 | 138 | 0.5 |
|  |  |  |  | 10 | 352 | 137 | 1 |
|  |  |  |  | 11 | 353 | 136 |  |

Table 13:Aggregate the results of table 6-12

|  |  | CORE | Positive | Ture positive | Negaitve | Ture Negative | P |
| --- | --- | --- | --- | --- | --- | --- | --- |
| AGE | ≥ | 8 | 209 | 214 | 54 | 49 | 0.063 |
|  | ＜70 | 6 | 236 | 240 | 110 | 106 | 0.125 |
| BMI | ＜25 | 9 | 261 | 263 | 103 | 101 | 0.5 |
|  | ≥25 | 6 | 188 | 191 | 57 | 54 | 0.25 |
| transverse diameter | ＜4.9 | 5 | 241 | 246 | 47 | 42 | 0.063 |
|  | ≥4.9 | 9 | 206 | 208 | 115 | 113 | 0.5 |
| anteroposterior diameter | ≥3.4 | 9 | 220 | 222 | 116 | 114 | 0.5 |
|  | ＜3,4 | 5 | 227 | 232 | 46 | 41 | 0.063 |
| cephalocaudal diameter | ＜4 | 5 | 237 | 241 | 50 | 46 | 0.125 |
|  | ≥4 | 9 | 204 | 206 | 111 | 109 | 0.5 |
| Lesion’s longest diameter | ＜1.5 | 7 | 219 | 224 | 85 | 80 | 0.063 |
|  | ≥1.5 | 5 | 225 | 230 | 80 | 75 | 0.063 |
| PSA | ≥20 | 2 | 96 | 101 | 24 | 19 | 0.063 |
|  | ＜20 | 9 | 351 | 353 | 138 | 136 | 0.5 |
| PI-RADS | 3 | 9 | 85 | 86 | 112 | 111 | 1 |
|  | 4 | 6 | 202 | 204 | 39 | 37 | 0.5 |
|  | 5 | 4 | 160 | 164 | 11 | 7 | 0.125 |

Table 14: Distribution range of Disease Risk Score (DRS)

| layer | PS value | |
| --- | --- | --- |
|  | Lower limit | Upper limit |
| 1 Min-P20 | 0 | 0.4961872 |
| 2, P20-P40, | 0.4961872 | 0.7849421 |
| 3,P40-P60, | 0.7849421 | 0.9023545 |
| 4, P60-P80, | 0.9636740 | 0.9636740 |
| 5 P80-Max. | 0.9636740 | 1 |
